# Supplementary material for: Organizing pneumonia of COVID-19: Time-dependent evolution and outcome in CT findings
Source: PLoS One. 2020 Nov 11;15(11):e0240347. doi: 10.1371/journal.pone.0240347 (PMC7657520; doi:10.1371/journal.pone.0240347)
Supplement: S1 Table — Abbreviations: GGO = ground glass opacity; with three signs = GGO, consolidation and linear opacity. (DOCX) [file pone.0240347.s001.docx]

**S1 Table. CT findings in COVID-19 patients with organizing pneumonia pattern**

| **CT findings** | **Count (percentage)** |
| --- | --- |
| **CT signs** |  |
| Pure GGO | 415(32.2%) |
| GGO and consolidation | 272(21.2%) |
| Pure consolidation | 148(11.5%) |
| Pure linear opacity | 24(1.9%) |
| GGO and linear opacity | 83(6.5%) |
| Consolidation and linear opacity | 116(9.0%) |
| With three signs | 227(17.7%) |
| Reversed halo sign | 28(2.2%) |
| Air bronchogram | 353(27.5%) |
| Crazy paving | 26(2.0%) |
| **Involvement of lung lobes** |  |
| Right upper lobe | 230(17.9%) |
| Right middle lobe | 193(15.0%) |
| Right lower lobe | 318(24.7%) |
| Left upper lobe | 251(19.5%) |
| Left lower lobe | 293(22.8%) |

Abbreviations: GGO = ground glass opacity; with three signs = GGO, consolidation and linear opacity.
