# Supplementary material for: Organizing pneumonia of COVID-19: Time-dependent evolution and outcome in CT findings
Source: PLoS One. 2020 Nov 11;15(11):e0240347. doi: 10.1371/journal.pone.0240347 (PMC7657520; doi:10.1371/journal.pone.0240347)
Supplement: S2 Table — Note: *Significance at P<0.017 with Bonferroni correction. Abbreviations: GGO = ground glass opacity; with three signs = GGO, consolidation and linear opacity. (DOCX) [file pone.0240347.s002.docx]

**S2 Table. CT findings during different time groups in COVID-19 patients with organizing pneumonia pattern**

| **CT findings** | **Day 0-7**  **(*n*=353)** | **Day 8-14**  **(*n*=421)** | **Day >14**  **(*n*=511)** | ***P* value** | **Day 0-7**  **vs. Day 8-14**  ***P* value** | **Day 8-14**  **vs. Day>14**  ***P* value** |
| --- | --- | --- | --- | --- | --- | --- |
| **CT signs** |  |  |  |  |  |  |
| Pure GGO | 146(41.4%) | 129(30.6%) | 140(27.4%) | **<0.001** | **0.002*** | 0.280 |
| GGO and consolidation | 60(17.0%) | 97(23.0%) | 115(22.5%) | 0.070 | **0.040** | 0.850 |
| Pure consolidation | 32(9.1%) | 47(11.2%) | 69(13.5%) | **0.040** | 0.340 | 0.280 |
| Pure linear opacity | 3(0.8%) | 4(1.0%) | 17(3.3%) | **0.005** | 0.990 | **0.030** |
| GGO and linear opacity | 18(5.1%) | 35(8.3%) | 30(5.9%) | 0.800 | 0.080 | 0.150 |
| Consolidation and linear opacity | 22(6.2%) | 36(8.6%) | 58(11.4%) | **0.009** | 0.220 | 0.160 |
| With three signs | 72(20.4%) | 73(17.3%) | 82(16.0%) | 0.110 | 0.280 | 0.590 |
| **Involvement of lung lobes** |  |  |  | **<0.001** | **0.010** | 0.251 |
| Number of affected lobes≤3 | 53(50.5%) | 36(33.0%) | 33(26.2%) |  |  |  |
| Number of affected lobes>3 | 52(49.5%) | 73(67.0%) | 93(73.8%) |  |  |  |
| **Total CT score** | 4.4±2.6 | 5.3±2.9 | 5.5±2.7 | **0.010** | **0.020** | 0.570 |

Note: *****Significance at *P*<0.017 with Bonferroni correction.

Abbreviations: GGO = ground glass opacity; with three signs = GGO, consolidation and linear opacity.
