# Supplementary material for: Organizing pneumonia of COVID-19: Time-dependent evolution and outcome in CT findings
Source: PLoS One. 2020 Nov 11;15(11):e0240347. doi: 10.1371/journal.pone.0240347 (PMC7657520; doi:10.1371/journal.pone.0240347)
Supplement: S3 Table — Note: Unless otherwise indicated, data are reported as the number of patients, with percentages in parentheses. a, data were reported as the mean ± standard derivation. (DOCX) [file pone.0240347.s003.docx]

**S3 Table. Comparisons of demographics and laboratory test between complete absorption and residual groups in COVID-19 patients with organizing pneumonia**

| **Characteristics** | **Complete absorption**  **(n=20)** | **Residual**  **(n=47)** | ***P* value** |
| --- | --- | --- | --- |
| **Age (year)^a^** | 34.9±9.0 | 47.9±13.7 | **0.001** |
| **Male sex** | 11(55.0%) | 9(45.0%) | 0.710 |
| **Comorbidity** | 3(15.0%) | 18(38.3%) | 0.110 |
| **Exposure history** |  |  | 0.410 |
| Recent travel to Wuhan | 13(65.0%) | 22(47.8%) |  |
| Contact with infected patient | 5(25.0%) | 15(32.6%) |  |
| Unknown exposure | 2(10.0%) | 9(19.6%) |  |
| **Initial symptom** |  |  |  |
| Fever | 17(85.0%) | 42(91.3%) | 0.450 |
| Cough | 8(40.0%) | 27(58.7%) | 0.160 |
| Expectoration | 3(15.0%) | 15(32.6%) | 0.240 |
| Fatigue | 3(15.0%) | 7(15.2%) | 0.980 |
| Chest tightness and/or breath shortness | 2(10.0%) | 6(13.0%) | 0.990 |
| Pharyngalgia | 2(10.0%) | 7(15.2%) | 0.570 |
| Muscle soreness | 2(10.0%) | 5(10.9%) | 0.990 |
| Headache | 1(5.0%) | 4(8.7%) | 0.990 |
| Nausea and/or vomiting | 0(0) | 1(2.2%) | 0.990 |
| Diarrhea | 0(0) | 2(4.3%) | 0.870 |
| No obvious symptoms | 1(5.0%) | 1(2.2%) | 0.990 |
| **Laboratory test on admission** |  |  |  |
| C-reactive protein (mg/L) |  |  | **0.009** |
| 0-10 | 13(68.4%) | 13(29.5%) |  |
| >10 | 6(31.6%) | 31(70.5%) |  |
| <0 | 0(0) | 0(0) |  |
| Percentage of lymphocytes (%) |  |  | 0.560 |
| 20-50 | 12(60.0%) | 23(50.0%) |  |
| >50 | 1(5.0%) | 1(2.2%) |  |
| <20 | 7(35.0%) | 22(47.8%) |  |
| Lymphocyte count (×10^9^/L) |  |  | 0.140 |
| 1.10-3.20 | 15(75.0%) | 25(55.6%) |  |
| >3.20 | 0(0) | 0(0) |  |
| <1.10 | 5(25.0%) | 20(44.4%) |  |
| Percentage of monocytes (%) |  |  | 0.630 |
| 3.0-10.0 | 13(65.0%) | 35(76.1%) |  |
| >10.0 | 6(30.0%) | 9(19.6%) |  |
| <3.0 | 1(5.0%) | 2(4.3%) |  |
| White blood cell count (×10^9^/L) |  |  | 0.710 |
| 3.5-9.5 | 14(70.0%) | 30(65.2%) |  |
| >9.5 | 1(5.0%) | 1(2.2%) |  |
| <3.5 | 5(25.0%) | 15(32.6%) |  |
| Alanine Aminotransferase (U/L) |  |  | 0.540 |
| 7-40 | 17(85.0%) | 34(75.6%) |  |
| >40 | 3(15.0%) | 9(20.0%) |  |
| <7 | 0(0) | 2(4.4%) |  |
| Aspartate Aminotransferase (U/L) |  |  | 0.840 |
| 13-35 | 16(80.0%) | 35(77.8%) |  |
| >35 | 4(20.0%) | 10(22.2%) |  |
| <13 | 0(0) | 0(0) |  |
| Creatine kinase (U/L) |  |  | 0.130 |
| 40-200 | 15(75.0%) | 39(86.7%) |  |
| >200 | 1(5.0%) | 4(8.9%) |  |
| <40 | 4(20.0%) | 2(4.4%) |  |
| Neutrophil percentage (%) |  |  | **0.020** |
| 40-75 | 14(70.0%) | 27(58.7%) |  |
| >75 | 2(10.0%) | 17(37.0%) |  |
| <40 | 4(20.0%) | 2(4.3%) |  |
| Hemoglobin (g/L) |  |  | 0.050 |
| 115-150 | 18(90.0%) | 36(78.3%) |  |
| >150 | 2(10.0%) | 1(2.2%) |  |
| <115 | 0(0) | 9(19.6%) |  |

Note: Unless otherwise indicated, data are reported as the number of patients, with percentages in parentheses.

^a^, data were reported as the mean ± standard derivation.
