# Supplementary material for: Organizing pneumonia of COVID-19: Time-dependent evolution and outcome in CT findings
Source: PLoS One. 2020 Nov 11;15(11):e0240347. doi: 10.1371/journal.pone.0240347 (PMC7657520; doi:10.1371/journal.pone.0240347)
Supplement: S4 Table — Abbreviations: GGO = ground glass opacity; with three signs = GGO, consolidation and linear opacity. (DOCX) [file pone.0240347.s004.docx]

**S4 Table. Comparisons of CT findings** **between complete absorption and residuals groups**

| **CT findings** | **Day 0-7** | | | **Day 8-14** | | |
| --- | --- | --- | --- | --- | --- | --- |
|  | **Complete absorption (*****n*=81)** | **Residual**  **(*n*=185)** | ***P* value** | **Complete absorption**  **(*n*=65)** | **Residual**  **(*n*=269)** | ***P* value** |
| **CT signs** |  |  |  |  |  |  |
| Pure GGO | 32(39.5%) | 83(44.9%) | 0.420 | 19(29.2%) | 74(27.5%) | 0.780 |
| GGO and consolidation | 14(17.3%) | 32(17.3%) | 0.990 | 22(33.8%) | 58(21.6%) | **0.040** |
| Pure Consolidation | 12(14.8%) | 11(5.9%) | **0.020** | 1(1.5%) | 37(13.8%) | **0.010** |
| Pure linear opacity | 0(0) | 1(0.5%) | 0.990 | 1(1.5%) | 2(0.7%) | 0.990 |
| GGO and linear opacity | 4(4.9%) | 8(4.3%) | 0.990 | 6(9.2%) | 27(10.0%) | 0.850 |
| Consolidation and linear opacity | 5(6.2%) | 8(4.3%) | 0.520 | 3(4.6%) | 21(7.8%) | 0.530 |
| With three signs | 14(17.3%) | 42(22.7%) | 0.320 | 13(20.0%) | 50(18.6%) | 0.790 |
| Reversed halo sign | 4(4.9%) | 8(4.3%) | 0.990 | 2(3.1%) | 9(3.3%) | 0.990 |
| Air bronchogram | 22(27.2%) | 60(32.4%) | 0.390 | 14(21.5%) | 91(33.8%) | 0.060 |
| Crazy paving | 0(0) | 1(0.5%) | 0.990 | 0(0) | 1(0.4%) | 0.990 |
| **Involvement of lung lobes** |  |  | 0.220 |  |  | **0.030** |
| Number of affected lobes≤3 | 11(61.1%) | 17(43.6%) |  | 9(60.0%) | 11(27.5%) |  |
| Number of affected lobes>3 | 7(38.9%) | 22(56.4%) |  | 6(40.0%) | 29(72.5%) |  |
| **Total CT score** |  |  | 0.100 |  |  | **0.010** |
| CT score≤4 | 13(72.2%) | 19(48.7%) |  | 12(80.0%) | 14(35.0%) |  |
| CT score>4 | 5(27.8%) | 20(51.3%) |  | 3(20.0%) | 26(65.0%) |  |

Abbreviations: GGO = ground glass opacity; with three signs = GGO, consolidation and linear opacity.
