# Supplementary material for: Organizing pneumonia of COVID-19: Time-dependent evolution and outcome in CT findings
Source: PLoS One. 2020 Nov 11;15(11):e0240347. doi: 10.1371/journal.pone.0240347 (PMC7657520; doi:10.1371/journal.pone.0240347)

**S1 Fig. ROC curve for total CT score to distinguish residuals group from complete absorption group at day 8-14**


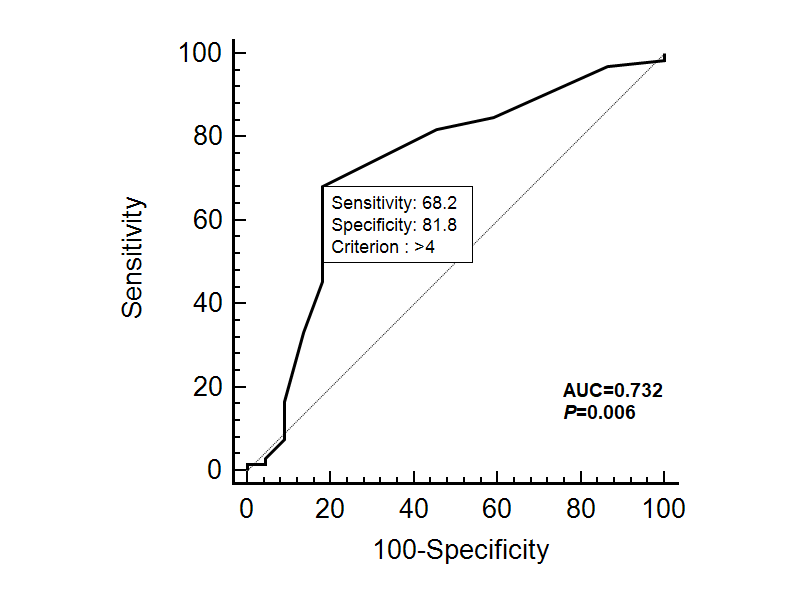

Supplement: S1 Fig — (DOCX) [file pone.0240347.s005.docx]
