# Supplementary material for: Organizing pneumonia of COVID-19: Time-dependent evolution and outcome in CT findings
Source: PLoS One. 2020 Nov 11;15(11):e0240347. doi: 10.1371/journal.pone.0240347 (PMC7657520; doi:10.1371/journal.pone.0240347)
Supplement: Attachment — Submitted filename: Response to Editor-0905.docx [file pone.0240347.s007.docx]

**Response to Editor’s Comments**

1) Please amend your current ethics statement to include the full name of the ethics committee/institutional review board(s) that approved your specific study.

Re to ED1: Thanks for your comments. We have revised the current ethics statement and included the full name of the ethics committee/institutional review board(s) as following: “ This multicenter study was launched by the First Affiliated Hospital of Xi’an Jiaotong University, and approved by all the multicenter institutions, including the First Affiliated Hospital of Xi’an Jiaotong University, the Eighth Hospital of Xi’an, Xi’an Chest Hospital, Ankang Center Hospital, Wuhan No.9 Hospital, Hanzhong Center Hospital, Baoji Center Hospital. For ethic issue, IRB protocol of XJTU1AF2020LSK-011 is multiple-center.” The above statement has been added at the beginning of the Methods section of our manuscript file.

2) We note that your Data Availability Statement reads, "Since this is multi-center cooperation data, we do not have full access to it, so we can only share part of the data from our hospital. All relevant data are within the manuscript and its Supporting Information files."

Could you please clarify whether "Supporting information.zip" contains your full Minimal Data Set, or whether the data from additional centers/hospitals is necessary for replication?

Re to ED2: Thanks for your suggestion. Due to the data privacy protection policy, we have tried our best to communicate with multi-center ethics review board and sorted minimal data set to support our conclusion. This minimal data set was from multiple hospitals and was able to replicate our conclusions.
